# Noninvasive prenatal testing for chromosome aneuploidies and subchromosomal microdeletions/microduplications in a cohort of 42,910 single pregnancies with different clinical features

**DOI:** 10.1186/s40246-019-0250-2

**Published:** 2019-11-29

**Authors:** Yibo Chen, Qi Yu, Xiongying Mao, Wei Lei, Miaonan He, Wenbo Lu

**Affiliations:** 1 Ningbo Women and Children Hospital, No.339, Liuting Street, Haishu District Ningbo, Ningbo, 315010 China; 2 CapitalBio Technology Inc., Beijing, 101111 China; 3 Beijing CapitalBio Medical Laboratory, Beijing, 101111 China

**Keywords:** Noninvasive prenatal testing (NIPT), Chromosome aneuploidies, Microdeletion/microduplication syndromes (MMS), Clinical features, Positive predictive value (PPV)

## Abstract

**Background:**

Since the discovery of cell-free DNA (cfDNA) in maternal plasma, it has opened up new approaches for non-invasive prenatal testing. With the development of whole-genome sequencing, small subchromosomal deletions and duplications could be found by NIPT. This study is to review the efficacy of NIPT as a screening test for aneuploidies and CNVs in 42,910 single pregnancies.

**Methods:**

A total of 42,910 single pregnancies with different clinical features were recruited. The cell-free fetal DNA was directly sequenced. Each of the chromosome aneuploidies and the subchromosomal microdeletions/microduplications of PPV were analyzed.

**Results:**

A total of 534 pregnancies (1.24%) were abnormal results detected by NIPT, and 403 pregnancies had underwent prenatal diagnosis. The positive predictive value (PPV) for trisomy 21(T21), trisomy 18 (T18), trisomy 13 (T13), sex chromosome aneuploidies (SCAs), and other chromosome aneuploidy was 79.23%, 54.84%, 13.79%, 33.04%, and 9.38% respectively. The PPV for CNVs was 28.99%. The PPV for CNVs ≤ 5 Mb is 20.83%, for within 5–10 Mb 50.00%, for > 10 Mb 27.27% respectively. PPVs of NIPT according to pregnancies characteristics are also different.

**Conclusion:**

Our data have potential significance in demonstrating the usefulness of NIPT profiling not only for common whole chromosome aneuploidies but also for CNVs. However, this newest method is still in its infancy for CNVs. There is still a need for clinical validation studies with accurate detection rates and false positive rates in clinical practice.

## Introduction

Since the discovery of cell-free fetal DNA (cffDNA) in maternal plasma in 1997 [1], it has opened up new approaches for non-invasive prenatal testing (NIPT). Since 2011, massively parallel sequencing (MPS) for fetal aneuploidies has become available in more than 60 countries. NIPT using cfDNA circulating in maternal blood has opened the door to early, accurate, and safe prenatal testing, and it has been available clinically for over 8 years [2]. Weighted pooled detection rates and false-positive rates for screening for trisomy 21, 18, 13, monosomy X, and other sex aneuploidies are reported at 99.2% (0.09%), 96.3% (0.13%), 91% (0.13%), 90.3% (0.23%), and 93% (0.14%) respectively [3]. A growing number of studies demonstrate that NIPT could reduce the incidence of unnecessary invasive procedures and iatrogenic fetal loss [4]. NIPT had many additional advantages over traditional biochemical and sonographic screening, such as higher sensitivities and specificities and ability to conduct NIPT at an earlier gestational age. In China, NIPT is recommended for screening trisomy 21 (T21), T18, and T13 for patients with high risk of serological screening results in the second trimester [5]. Now, more and more pregnant women are willing to choose NIPT [6, 7].

This newest method of prenatal screening has other applications, including screening for microdeletion/microduplication syndromes (MMS) caused by copy-number variants (CNVs) < 10 Mb. MMS are individually rare, but together account for 1–2% of all newborn congenital abnormalities and often resulting in a severe burden for families and society. More recently, further development and expansion of NIPT has focused on MMS, such as Hu et al. [8] and Liang et al. [9] demonstrated NIPT performed well in some MMS.

However, there are many problems and challenges in clinical practice, and extensive validation is needed to determine accurately its detection rate and false-positive rate. The study’s objective is to review the efficacy of NIPT as a screening test for aneuploidies and CNVs in 42,910 single pregnancies.

## Results

### Patient characteristics

From April 2015 to December 2018, a total of 42,931 maternal blood samples were collected from Ningbo Women and Children Hospital in China. In 21 cases, detection failed, with a failure rate of 0.05%. Thus, the total sample included in this study was 42,910. The pregnancy gestations were 12^+0^~26^+6^, the years of age were 18–49, and there were 10,742 women with advanced maternal age (age ≥ 35 years). Clinical characteristics of the 42,910 cases are shown in Table 1. Of those 42,910 samples, there were 348 pregnant women who needed resampling due to low fetal DNA concentration in plasma; thus, the resampling rate was 0.81% (348/42910), and all the resamplings obtained a NIPT result (Table 1).

**Table 1:** Clinical characteristic of pregnant women undergoing NIPT

Gestational age at NIPT (weeks)	No./*N* = 42910	Rate (%)
12~15^+6^	5535	12.90
16~19^+6^	24759	57.70
20~23^+6^	10513	24.50
24~26^+6^	2103	4.90
Clinical features	No.	Rate (%)
Fetal structural abnormalities by B-ultrasound	202	0.47
Increased NT	5749	13.4
Other^a^	12	0.03
High risk of serological screening	2318	5.40
Critical risk of serological screening	15863	36.97
Advance maternal age (≥ 35 years)	10742	25.03
No clinical indications	8024	18.70

^a^Patients with interventional surgery contraindications: reoperative infection, placenta previa, placental bleeding, poor pregnancy history

### Prenatal test results of total pregnant

Before NIPT, pregnant women conventionally conducted screening test involving fetal ultrasonography (including color ultrasound and three dimension color ultrasound) and maternal serum biomarkers determination. Ultrasonography showed that 202 (0.47%) fetuses were structurally abnormal, and there were 5749 (13.4%) fetuses with an increased NT (NT ≥ 3 mm). Maternal serum biomarkers determination suggested there were 2318 (5.4%) high risk pregnancies and 15,863 (36.97%) critical risk pregnancies; 8024 (18.70%) pregnancies had no clinical indications (Table 1).

### NIPT results for T21, T18, T13, and SCAs

**Fig. 1:**
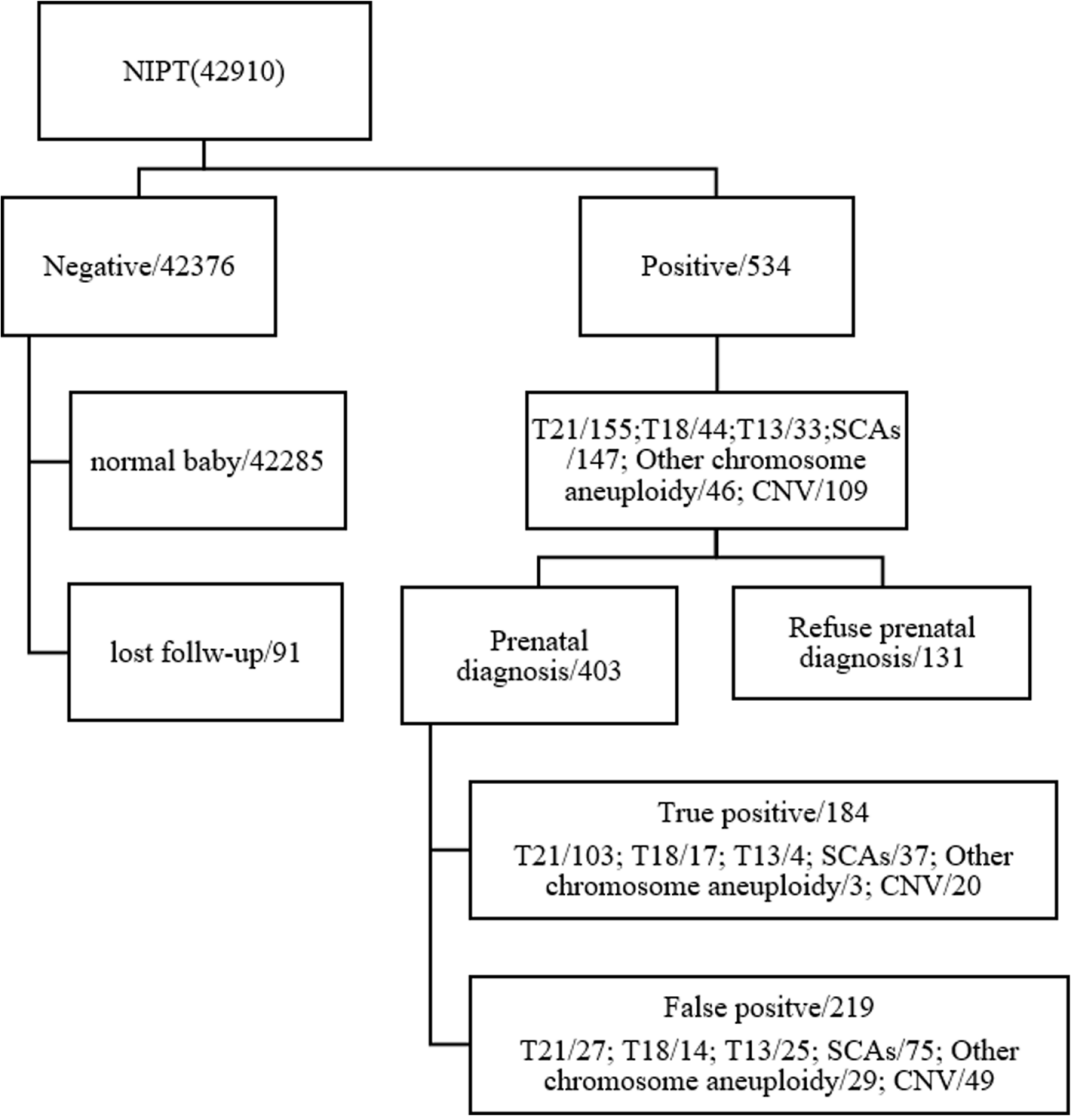
Flowchart of non-invasive prenatal test (NIPT) results and clinical outcome of pregnant women

The flowchart is shown in Fig. 1. A total of 42,931 samples were recruited with 42,910 NIPT results, including 534 (1.24%) abnormal results. Of these 534 cases, there were 155 of trisomy 21 (T21), 44 of T18, 33 of T13, 147 of sex chromosome abnormalities (SCAs), 46 of other chromosome aneuploidy (except T21, T18, T13, and sex chromosome aneuploidy), and 109 of CNVs.

Karyotype was obtained to verify the abnormal results of the NIPT predictions. The total abnormal results of T21, T18, T13, and SCAs were 379. Of these 379, there were 302 cases underwent prenatal diagnostic testing, which confirmed 161 cases of true positive (103 cases of T21, 17 of T18, 4 of T13, 37 of SCAs) and 141 cases of false positive (FP). Moreover, the positive predictive value (PPV) for each test was assessed. For trisomy 21, the PPV was 79.23%, for trisomy 18, 54.84%, for trisomy 13, 13.79%, and for SCAs, 33.04% (Table 2 and Fig. 1).

**Table 2:** Performance of non-invasive prenatal testing (NIPT) chromosome aneuploidy

NIPT	Trisomy 21	Trisomy 18	Trisomy 13	SCAs	Other chromosome aneuploidy	CNVs	Total
Positive	155	44	33	147	46	109	534
TP	103	17	4	37	3	20	184
FP	27	14	25	75	29	49	219
PPV`	79.23%	54.84%	13.79%	33.04%	9.38%	28.99%	45.66%

*TP* true positive, *FP* false positive, *PPV* positive predictive value, *SCAs* sex chromosomal aneuploidies, *CNV* copy-number variations

### NIPT results for CNVs and other chromosome aneuploidies

Besides, we have also analyzed the CNVs and other chromosome aneuploidies, because this technology is genome-wide sequencing. The total cases of CNVs abnormal results are 109, including 20 true positive cases, 49 false positive cases, and 50 unverified cases. CNV number and size on each chromosome was assessed. CNVs were categorized into three groups according to length: CNVs ≤ Mb, CNVs within 5–10 Mb, and CNVs > 10 Mb. The PPV for each group was also assessed. The total PPV for CNVs was 28.99%. The PPV for CNVs ≤ 5 Mb is 20.83%, for CNVs within 5–10 Mb is 50.00%, for CNVs > 10 Mb is 27.27% respectively. The total cases of other chromosome aneuploidy are 46, including 3 true positive, 29 false positive, and 14 unverified cases. The PPV for other chromosome aneuploidy was 9.38%. In other chromosome aneuploidy, Chr7 aneuploidy is the largest group. All of Chr7 aneuploidy predicted by NIPT are trisomy 7, but all verified patients (total number is 9) were confirmed to be false positives (Table 3 and Table 4).

**Table 3:** The size and number of CNVs and other chromosome aneuploidies on each chromosome

Chr.	CNVs length			CNVs				Other chromosome aneuploidies			
	≤ 5 Mb	Within 5–10 Mb	> 10 Mb	NIPT positive	NIPT true positive	NIPT false positive	Unverified	NIPT positive	NIPT true positive	NIPT false positive	Unverified
Chr1	0	0	1	1	1	0	0	/	/	/	/
Chr2	0	0	2	2	0	2	0	/	/	/	/
Chr3	0	0	2	2	0	0	2	2	0	2	0
Chr4	0	1	2	3	1	2	0	1	0	0	1
Chr5	0	1	2	3	0	1	2	1	1	0	0
Chr6	0	1	0	1	0	1	0	/	/	/	/
Chr7	3	1	6	10	1	8	1	14	0	9	5
Chr8	1	1	4	6	0	1	5	5	0	3	2
Chr9	0	0	4	4	0	1	3	2	0	1	1
Chr10	0	0	3	3	0	3	0	1	0	0	1
Chr11	0	0	5	5	1	2	2	1	0	1	0
Chr12	/	/	/	/	/	/	/	1	0	1	0
Chr13	2	2	4	8	2	3	3	/	/	/	/
Chr14	0	0	2	2	1	1	0	3	0	2	1
Chr15	2	1	0	3	1	1	1	2	1	1	0
Chr16	2	0	1	3	0	3	0	4	0	4	0
Chr17	3	1	0	4	0	3	1	/	/	/	/
Chr18	4	2	7	13	4	3	6	/	/	/	/
Chr19	/	/	/	/	/	/	/	/	/	/	/
Chr20	0	1	2	3	0	0	3	5	1	3	1
Chr21	0	0	1	1	1	0	0		/	/	/
Chr22	9	0	5	14	2	8	4	4	0	2	2
X or Y	11	5	2	18	5	6	7	/	/	/	0
Total	37	17	55	109	20	49	40	46	3	29	14

**Table 4:** The PPVs according to different CNV sizes

CNV size	NIPT positive	TP	FP	Unverified	PPV (%)
≤ 5 Mb	37	5	19	13	20.83
Within 5–10 Mb	17	6	6	5	50.00
> 10 Mb	55	9	24	22	27.27
Total	109	20	49	40	28.99

### Different PPV according to pregnancies characteristics

Different PPVs of NIPT according to pregnancies characteristics are shown in Table 5. The total PPV of T21 is 79.23%, the PPV of T21 fetuses in women of advanced maternal age is 89.29%, in high-risk of serological screening group is 86.67%, and in critical risk of serological screening group is 71.74%, and the PPV of NIPT in increased NT group is the highest, which is 100%. Similarly, the PPV in increased NT group is also the highest in predicting SCAs fetuses. It is worth noting that the PPVs of T18 fetuses in fetal structural abnormalities by B-ultrasound group, increased NT group, and high risk of serological screening are all 100%, while in predicting T13 and CNVs in fetuses, the PPV of high risk of serological screening group and fetal structural abnormalities by B-ultrasound group is the highest respectively.

**Table 5:** Different PPVs according to pregnancies characteristics

Clinical features	PPV of T21 (%)	PPV of T18 (%)	PPV of T13 (%)	PPV of SCAs (%)	PPV of other chromosome aneuploidy (%)	PPV of CNVs (%)
Fetal structural abnormalities by B-ultrasound	0	100	0	0	0	100.00
Increased NT	100	100	/	50.00	0	37.50
Other^a^	/	/	/	/	/	0.00
High risk of serological screening	86.67	100	100	28.57	12.50	11.11
Critical risk of serological screening	71.74	33.33	9.09	28.57	0	50.00
Advanced maternal age (≥ 35 years)	89.29	60.00	9.09	30.77	15.38	5.26
No clinical indications	33.33	0	25.00	35.71	0	41.67
Total	79.23	54.84	13.79	33.04	9.09	28.99

“/” indicates no data

^a^Patients with interventional surgery contraindications: reoperative infection, placenta previa, placental bleeding, poor pregnancy history.

## Discussion

NIPT has been widely used for prenatal screening of T21, T18, and T13 in the last few years. But, up to now, it is still lacking large scale clinical studies focused on the efficiency in subchromosomal copy number variations (CNVs), typically less than 5 Mb in size [8, 9]. Besides, there are really some concerns on the clinical performance [10, 11]. Therefore, we hope that the present research including 42,910 cases can provide data support for these issues.

We used positive predictive value (PPV) to evaluate NIPT in this study. The PPV for T21 was 79.23%, and for T18, T13; SCAs were 54.84%, 13.79%, 33.04% respectively. Besides, we also analyzed the PPV of other chromosome aneuploidy and CNVs. The PPV for other chromosome aneuploidy was 9.38%, and for CNVs was 28.99%. In several recent studies, the PPV range of T21 was 65–94%, T18 was 47–85%, and T13 was 12–62% [12–14]. Our results fall within this range. Interestingly, the PPV for CNVs was 28.99%, obviously higher than that of T13. Previous clinical validation studies reported a variable performance for detection of specific MMS, with only low to moderate positive predictive values (PPVs) [9].

Recently, more relaxed guidelines have been suggested whereby screening for MMS can be performed routinely for younger women where microdeletions are more frequent than aneuploidies [15]. Based on its performance in this retrospective study of over 42,000 pregnancies, NIPT displays the hallmarks of a screening method suitable for MMS caused by CNVs. The PPV for CNVs within 5–10 Mb is the highest (50.00%) in this study, and PPV for CNVs ≤ 5 Mb is the lowest (20.83%). In Liang’s paper (ref. [9]), the PPV for CNVs > 10 Mb (32%) and CNVs < 10 Mb (19%) also were low but reasonable, indicating possible sufficient sensitivity and specificity of the test for potential screening of genome-wide fetal CNVs. PPV depends not only on the sensitivity and specificity of the assay, but also on the prevalence of the disease [16]. The PPV for CNVs < 10 Mb is 31% in this study (data not shown in the table, PPV = (5 + 6)/[(5 + 6) + (19 + 6)]), which is much higher than Liang’s paper. In addition, a previous study reported an overall PPV for CNVs of 9.2% [17], and the PPV in our study is much higher than that.

The PPVs for other chromosome aneuploidy were lower at 9.38% and similar to those reported also in Liang’s paper (ref. [9]). The reason is that these aneuploidies are less prevalent and many of them have high rates of confined placental mosaicism (CPM). NIPT is performed using cell-free fetal DNA, and the primary source of cell-fetal DNA in the maternal circulation is thought to be apoptosis of placental cells from the cytotrophoblast [18], which is not always representative of the fetus. A situation in which a chromosomal abnormality occurs only in the placenta but not in the fetus is known as CPM [19] where observations of the incidence are around 1–2% [20]. NIPT is a screening test. For pre-counseling for NIPT, women who choose should be well informed about the accuracy, reliability, false positive, and false negative rates. For post counseling, in regard to current NIPT guidelines, ACMG is strongly suggested to confirm by invasive prenatal diagnosis for all positive findings [21]. In addition, all women who carried a fetus suspected of having a confirmed pathogenic or likely pathogenic fetal chromosome anomaly were scheduled for a genetic counseling session to discuss pregnancy management options.

We have also made further thought about the different PPV of NIPT according to pregnancies characteristics, and the results in this section need more clinical data support. Different pregnancies characteristics show different PPV, and the PPV of NIPT is the highest for T21 and is much lower for other aneuploidies [22]. Advanced maternal age (usually ≥ 35 years) is a high risk factor for T21. So, PPV in advanced maternal age is much higher than no clinical indications group. And PPV in high risk of serological screening group is higher than in critical risk group, which is consistent with Yu’s paper [23], while advanced maternal age may not be a risk indication for T18 and T13. Similarly, unlike aneuploidy, the most common CNVs are not related to maternal age, so the PPV for advanced maternal age does not show a higher value.

CNVs have become increasingly recognized as significant contributors to human diseases [24], which are present in approximately 1.7% of all structurally normal pregnancies [25]. Chromosomal microarray analysis (CMA) is a powerful tool for the detection of invisible small chromosomal deletions or duplications and was recommended as a first-tier diagnostic tool for some patients with well-defined syndromes [26, 27]. However, CMA has many limitations. Because sampling of CMA requires invasive testing and invasive test are associated with risk [28], such as miscarriage, abortion, and intrauterine infection [29], or because it may identify variants of uncertain significance, some women may decline it. It has shown that NIPT detected subchromosomal copy-number variants (CNVs) performed well in some MMS [30], and in recent years, there have been quite a few reports on NIPT expanded for MMS [8, 9, 31]. But, NIPT is a screening test, there still a need for clinical validation on its accurate detection rates and false positive rates with a large number of clinical samples.

In the present study, follow-up is for negative results. According to the guideline of National Health Commission of the People’s Republic of China, follow-up began at week 12 after delivery. Follow-up content should include the pregnancy outcomes of the subjects and the health of the newborn. The main follow-up content for newborn is whether the newborn is a T21 or T18 or T13 patient. Our follow-up began from 3 months after birth and strictly followed the national guideline in this study. At the time of follow-up, parents complained of neonatal with birth defects should go further genetic diagnosis. Besides, for CNVs, we have discussed the positive results, and we hope this study could provide validation for NIPT as a screening test for aneuploidies and CNVs.

## Conclusion

In conclusion, this study involved a large prospective group of pregnant women with different clinical characters. The data have potential significance in demonstrating the usefulness of NIPT profiling not only for common whole chromosome aneuploidies but also for CNVs. However, this newest method is still in its infancy for CNVs. There is still a need for clinical validation studies with accurate detection rates and false positive rates in clinical practice.

## Materials and methods

### Patients

Pregnant women were collected consecutively. From April 2015 to December 2018, pregnant women who came to Ningbo Women and Children Hospital for prenatal examination were recruited. A total of 42,910 pregnant women were recruited. Prior to blood sampling, a signed consent form was obtained from each participant. Inclusion criteria were as follows: (1) pregnancy gestation period between 12^+0^~26^+6^, (2) single pregnancy, and (3) body mass index (BMI) < 100. Exclusion criteria were as follows: (1) pregnant women with chromosomal abnormalities, (2) multiple pregnancy, (3) pregnant women who have received stem cell therapy and transplant surgery, (4) received allogeneic blood products within 1 year, and (5) received immunotherapy within 4 weeks.

#### Serological screening and ultrasonography

We used combined first trimester screening from 11 weeks to 13^+6^ weeks, and serological screening test was detected: the concentrations of AFP, free bHCG, and free E3 were detected by time-resolved immunofluorescence assay. NT was measured by a trained sonographer according to the Fetal Medicine Foundation protocol [32]. The risk values were calculated by Lifecycle software (4.0): high risk, T21 > 1/300, T18 > 1/350; intermediate risk, T21 1/300 to 1/1000, T18 1/350 to 1/1000; defining maternal age (AMA), maternal age ≥ 35 years [23]; and defining NT ≥ 3 mm as increased NT [33].

#### Sequencing

Maternal peripheral blood (5 ml) was collected in an ethylenediaminetetraacetic acid (EDTA) tube at a gestational age of 12^+0^ to 26^+6^ weeks. The blood sample was stored at 4 °C immediately after collection. Plasma was isolated within 8 h with a two-step centrifugation protocol according to the previous description (ref. [6]). The cell-free DNA extraction, library construction, sequencing, and bioinformatics analysis were performed according to the previous study (ref. [6]). High-throughput sequencing of fetal-free DNA fragments uses JingXin BioelectronSeq 4000 System (CFDA registration permit NO. 20153400309) semiconductor sequencer. Sequencing reads were filtered and aligned to the human reference genome (hg19). A combined GC correction and Z-score testing methods were used to identify fetal autosomal aneuploidy. Here, each chromosome with an absolute value of the Z-score greater than 3 was marked with chromosome aneuploidies or microdeletions/microduplications.

#### Karyotype analysis and amniotic fluid puncture

Women with positive NIPT results were recommended to receive karyotype analysis in amniotic fluid for further validation. The amniotic fluid puncture was performed as routinely described. The karyotype analysis was performed according to the International System for Human Cytogenetic Nomenclature guidelines [34].

#### Follow-up for negative cases

Follow-up investigation was performed to NIPT negative cases. According to the guideline of National Health Commission of the People’s Republic of China, follow-up began at week 12 after delivery. Follow-up content should include the pregnancy outcomes of the subjects and the health of the newborn. The main follow-up content for newborn is whether the newborn is a T21 or T18 or T13 patient. Our follow-up began from 3 months after birth and strictly followed the national guideline in this study. At the time of follow-up, parents complained of neonatal with birth defects should go further genetic diagnosis. Patients lost to follow-up were excluded from the analysis.

#### Statistical analysis

Statistical analysis was used SPSS 20.0 software. Measurement data were expressed as mean ± standard deviation (*x* ± SD), count data adoption rate (%), and positive predictive value = true positive number/all positive cases.

## Data Availability

The datasets used and/or analyzed during the current study are available from the corresponding author on reasonable request.

## Abbreviations

cfDNA: Cell-free DNA
CMA: Chromosomal microarray analysis
CNVs: Copy-number variants
MMS: Microdeletion/microduplication syndromes
NIPT: Non-invasive prenatal testing
NT: Nuchal translucency
PPV: Positive predictive value
